# Physical literacy conceptions in teacher training: a longitudinal study

**DOI:** 10.3389/fpsyg.2025.1699089

**Published:** 2025-12-04

**Authors:** Ariadna Hernaiz-Sánchez, Miguel A. González-Valeiro, Óscar Romero-Chouza, María A. Fernández-Villarino

**Affiliations:** 1 Departamento de Educación e Innovación Educativa, Facultad de Ciencias Jurídicas, Educación y Humanidades, Universidad Europea de Madrid, Campus de Villaviciosa, Madrid, Spain; 2 Department of Physical Education and Sport, University of A Coruña, A Coruña, Spain; 3 CIDEGA, Faculty of Education Sciences and Sport, University of Vigo, Pontevedra, Spain

**Keywords:** physical literacy, physical education, teacher training, beliefs and attitudes, higher education

## Abstract

**Introduction:**

Physical literacy (PL) embodies the motivation, confidence, physical competence, knowledge, and understanding necessary to sustain physical activity throughout life. Understanding how future physical education (PE) teachers conceptualize PL is essential for its effective implementation in schools. This longitudinal study examined the evolution of trainee teachers’ conceptions of PL across 5 years of initial teacher education in Spain.

**Methods:**

A total of 397 students (76% male; 22.6 ± 3.8 years) from two universities participated, with data collected at three time points: the first and final years of the bachelor’s degree in Physical Activity and Sport Sciences and the final year of the master’s degree in Teacher Training. Responses to the open question “What is a physically literate student?” were coded into 13 conceptual categories and analysed through two-step cluster analysis, multinomial logistic regression, and chi-square tests (*p* < 0.05).

**Results:**

Three profiles emerged: (1) an Enjoyment-Oriented group focused on participation and enjoyment in physical activity; (2) a Performance-Centred group emphasizing motor skills, physical fitness, and sport competence; and (3) a Competence-Driven group highlighting motor competence and active lifestyles. Over time, students’ conceptions shifted significantly (*χ*^2^ = …, *p* < 0.05) from enjoyment and personal competence towards motor competence and performance-related attributes, with lifestyle habits and maternal education predicting cluster membership.

**Discussion:**

These findings indicate that initial teacher education may foster more performance-oriented understandings of PL, underscoring the need for balanced curricula that integrate physical, cognitive, and affective dimensions of physical literacy.

## Introduction

Physical literacy represents a central perspective for contemporary Physical Education (PE) as it encapsulating the essence of what quality PE aims to achieve (Tremblay and Lloyd, 2010; Godbout, 2021; Stoddart and Humbert, 2021). It is widely understood as a lifelong, person-centred process that integrates physical, cognitive, and affective dimensions to support active and healthy living. Each individual develops PL according to personal and contextual factors, pursuing continuous growth rather than a fixed endpoint (Durden-Myers et al., 2018a; Mandingo et al., 2009). In this sense, fostering PL contributes to the holistic development and wellbeing of learners, one of the key challenges of 21st-century (López-Pastor et al., 2016).

Despite its relevance, the literature reveals persistent inconsistencies and diverse interpretations of the term (Martins et al., 2021). Among the available frameworks, this study adopts Whitehead’s definition of physical literacy as “the motivation, confidence, physical competence, knowledge and understanding to maintain physical activity throughout life” (Whitehead, 2019, 2021). This definition was chosen because it offers a comprehensive, philosophical, and educational foundation that emphasises embodiment, motivation, and reflection—key aspects when exploring teachers’ conceptions. In contrast, other frameworks such as the Canadian consensus definition or the Australian physical literacy framework focus more heavily on assessment and policy implementation. Whitehead’s holistic approach aligns with the pedagogical mission of PE teacher education and provides an appropriate conceptual lens for analysing future teachers’ beliefs about what it means to be physically literate.

Teachers play a decisive role in promoting PL (Carreiro-da-Costa, 2019; Whitehead, 2019). Their conceptions influence curriculum delivery, learning experiences, and the capacity of students to sustain active lifestyles (Lloyd et al., 2010). However, existing research suggests that many teachers are not fully familiar with the concept or interpret it narrowly in terms of performance and fitness (Durden-Myers et al., 2018b; Robinson et al., 2018). Recent studies highlight the need to strengthen pre-service teachers’ understanding of PL (Durden-Myers and Keegan, 2019; Stoddart and Selanders, 2022) as teacher education is often considered the starting point for successful implementation (Edwards et al., 2019). Nevertheless, most empirical investigations to date have been cross-sectional and descriptive, capturing teachers’ conceptions at a single point in time (Robinson et al., 2018; Stoddart and Selanders, 2022). Very few studies have examined how these conceptions evolve throughout the full initial teacher training process, which typically spans several academic stages. Likewise, little is known about how contextual variables, such as personal physical activity habits, socioeconomic background, or academic environment, shape these conceptions. This lack of longitudinal evidence represents a significant gap in the literature.

Addressing this gap is especially relevant in the Spanish educational context, where PL is still an emerging concept and often overlaps with the traditional notion of the “physically well-educated student” (Robinson and Randall, 2017). Spanish physical education is guided by official curricula that emphasize motor skill development, inclusive participation, and health promotion. Integrating PL into this framework aligns with national educational goals while also broadening the perspective to include motivation, confidence, and lifelong engagement. Understanding cultural and curricular determinants is therefore crucial to appreciate why Spanish trainee teachers should adopt the concept of PL and how it complements the local PE tradition. Examining the evolution of PL conceptions in Spanish pre-service teachers provides both a national contribution and an international comparison with the more established English-speaking research tradition.

Accordingly, the present study seeks to advance understanding of how conceptions of physical literacy develop during teacher education. Specifically, it aims to answer the following research questions:

1. What profiles of physical literacy conceptions exist among trainee physical education teachers?
2. How do these conceptions evolve across the 5 years of initial teacher training (bachelor’s and master’s degrees)?
3. Which contextual variables (e.g., physical activity habits, socioeconomic background) predict membership in particular conception profiles?

By addressing these questions, this study contributes novel longitudinal evidence on how future teachers conceptualize physical literacy, providing insights that can inform curriculum design and professional development in physical education teacher training.

Thus, the objectives set for this study were:

- To establish profiles of trainee teachers’ ideas about physical literacy.
- To identify the role of initial teacher training in the evolution of their ideas about physical literacy.

## Materials and methods

### Participants

The sample of this study consisted of 397 students, 303 male and 94 female students (22.6 ± 3.8 years of age) of the bachelor’s degree in physical Activity and Sport Sciences and the master’s degree in Teacher Training in two universities in north-western Spain. A convenience sampling approach was adopted. The two universities were selected because they offer comparable degree structures and collaborate in joint research programmes on Physical Education. All first-year students enrolled in the Bachelor’s degree were invited to participate. Participation was voluntary, and no academic credit or incentives were provided. Prior to data collection, participants received written information about the study aims, confidentiality procedures, and their right to withdraw at any time without consequence. Three data collections were conducted:

Time 1 (T1)—first-year undergraduate students (*n* = 397)

Time 2 (T2)—final-year undergraduate students, 4 years later (*n* = 321; attrition = 19.1%)

Time 3 (T3)—master’s students 1 year after graduation (*n* = 248; cumulative attrition = 37.5%)

Attrition was primarily due to study withdrawal, exchange programmes abroad, or failure to complete the questionnaires. Attrition analyses (Chi-square tests) showed no significant differences by gender, university, or initial cluster membership (*p* > 0.05).

The sample presented a marked gender imbalance (76.3% male). This reflects the typical gender composition of these programmes in Spain.

Table 1 describes the data on the distribution of the sample according to course, gender and university.

**Table 1:** Description of the sample.

	Women *n* = 94						Men *n* = 303					
	First year		Last year		Master		First year		Last year		Master	
	*N*	%	*N*	%	*N*	%	*N*	%	*N*	%	*N*	%
University of Vigo	32	8.6	19	4.7	5	1.2	47	11.8	53	13.3	10	2.5
University of A Coruña	18	4.5	15	3.7	5	1.2	92	23.1	82	20.6	19	4.7
Total	50	13.1	34	8.3	10	2.4	139	34.9	135	33.9	29	7.2

### Procedure

Given the longitudinal nature of the study, three data collection sessions were carried out. The initial data collection was carried out with students in the first year of the bachelor’s degree in physical Activity and Sport Sciences at the Universities. Four years later, a second data collection was carried out, when the same students were in the last year of their degree studies. They completed the questionnaire in the last week of the course. One year later, the last data collection was carried out with those students who decided to take the master’s degree in Teacher Training, thus completing the training cycle. Changes in the sample size from one data collection to another were due to reasons beyond the control of the research, such as students dropping out of their studies, stays abroad or failing subjects. In this way, we analysed the entire initial teacher training process that provides the qualifications necessary for professional practice in the context of Physical Education, monitoring the sample ideas throughout the entire initial training process.

Prior to data collection, the management of the two universities were informed and authorization was requested, and informed consent was sought from the participants. This study was conducted in line with ethical standards in sport science (Harriss and Atkinson, 2015) and was approved by the ethics committee of the authors ‘university before recruitment (University of Vigo, Ethical Application Ref: UV-13-170123).

### Research design and instrument

In order to analyse the ideas of future teachers throughout their initial training (bachelor’s degree in physical Activity and Sport Sciences and master’s degree in Teacher Training) on their conceptions of Physical Literacy, data were collected using the Professional Socialisation in Physical Education Questionnaire (Hernaiz-Sánchez et al., 2021), a validated instrument designed to assess conceptions and professional trajectories in Physical Education. The questionnaire comprises three dimensions with 36 items in total:

1. Personal data (6 items): age, gender, university, year, and physical activity habits.
2. Biographical characterisation (12 items): socioeconomic level, parental occupation, previous sport/PE experiences, and academic performance.
3. Conceptions of Physical Literacy (18 items): a combination of Likert-scale statements (1 = strongly disagree to 5 = strongly agree) and two open-ended questions exploring participants’ understanding of physical literacy and its relation to PE.

The questionnaire presents differences in the versions used for bachelor’s and master’s students, the latter omitting questions already answered in the previous questionnaire and adding new questions on the master’s training process.

### Variables

The key analytical variable derived from the open-ended question “What is a physically literate student?” Responses were analysed qualitatively and then coded quantitatively into 13 conceptual categories previously established during questionnaire validation (Hernaiz-Sánchez et al., 2021). Three independent researchers, experts in Physical Education pedagogy, coded all responses. Inter-rater reliability was verified using Cohen’s Kappa (*κ* = 0.87), indicating strong agreement. Discrepancies were resolved through discussion and consensus. Each participant was assigned to one primary category corresponding to the dominant idea expressed. The coding categories are available in Table 2.

**Table 2:** Categories of response to the research question.

Category 1	*Overall motor competence*: all the answers that talk about motor skills.
Category 2	*Sport competence*: all the answers that talk about sports skills.
Category 3	*Physical fitness*: all the answers that talk about physical fitness.
Category4	*Physical competence*: all the answers that talk about the attitudes related to physical abilities.
Category 5	*Sports-related physical fitness*: all the answers that talk about one or more sports.
Category 6	*Health-related physical fitness*: all the answers that talk about health.
Category 7	*Active lifestyle*: all the answers that talk about the importance of a healthy lifestyle.
Category 8	*Knowledge*: all the answers that talk about knowledge in general.
Category 9	*Sports knowledge*: all the answers that talk about knowledge related to sport.
Category 10	*Health-related knowledge*: all the answers that talk about kn0wledge related to health or physical condition.
Category 11	*Self-awareness*: all the answers that talk about knowledge related to one’s own body.
Category 12	*Personal competence*: all the answers that talk about the values of physical activity.
Category 13	*Attitudes/enjoyment*: all the answers that talk about positive feelings towards physical activity.

The main variable was the categorical classification of conceptions of physical literacy (13 categories). Contextual variables used as predictors or controls included: gender, university, academic year, previous PE experiences (yes/no), socioeconomic status (high/medium/low), weekly physical activity (hours), body mass index (BMI), parental occupation, perceived quality of PE received (poor–very good), and academic level (poor–very good).

### Statistical analysis

All analyses were conducted using IBM SPSS Statistics v25.0 (MacOS). The significance level was set at *p* < 0.05.

To identify natural groupings of students based on their conceptualisations of physical literacy, a Two-Step Cluster Analysis was performed using the 13 response categories. The analysis employed the log-likelihood distance measure (appropriate for categorical data) and the Bayesian Information Criterion (BIC) to determine the optimal number of clusters. The final three-cluster solution was selected based on the smallest BIC value and theoretical interpretability.

Cluster stability was assessed through split-sample validation (70% training, 30% testing subsample), which yielded a Cramer’s *V* = 0.81, indicating high consistency of cluster assignment.

A multinomial logistic regression model was then used to examine the influence of contextual variables on cluster membership. This model was chosen because the dependent variable (cluster) was nominal with three levels. Predictors were entered simultaneously (Enter method) to avoid the biases associated with stepwise selection.

To examine the evolution of conceptions across the three time points (T1–T3), Chi-square tests of independence were performed for each of the 13 categories. When expected frequencies were <5, categories were collapsed to meet test assumptions.

Effect sizes were calculated using Cramer’s *V*, and results were interpreted following conventional benchmarks (0.10 = small, 0.30 = medium, 0.50 = large).

## Results

To address the research objectives, the analyses were carried out in three stages:

- a cluster analysis to identify distinct profiles of conceptions about physical literacy,
- a multinomial logistic regression to determine which contextual variables predict cluster membership, and.
- a longitudinal analysis to examine the evolution of these conceptions over the 5 years of initial teacher training.

### Cluster profiles

The two-step cluster analysis produced a three-cluster solution that met the criteria for optimal fit based on the Bayesian Information Criterion (BIC) and cluster cohesion/separation ratio. The three profiles represented differentiated conceptual orientations among students regarding what defines a physically literate learner. Cluster sizes were as follows: Cluster 1 = 142 students (35.8%), Cluster 2 = 168 students (42.3%), and Cluster 3 = 87 students (21.9%).

Table 3 presents the percentage distribution of affirmative responses within each cluster across the 13 categories. As the coding derived from open-ended responses, students could contribute to more than one category.

**Table 3:** Percentages of affirmative responses in each cluster.

Category of response	Cluster 1 *n* = 185	Cluster 2 *n* = 131	Cluster 3 *n* = 79
Overall motor competence	0.5	15.9	10.0
Sports competence	0.5	15.2	0.0
Physical fitness	0.0	18.2	0.0
Physical competence	1.1	18.2	8.8
Sports-related physical fitness	4.3	0.8	0.0
Health-related physical fitness	2.7	2.3	1.3
Active lifestyle	38.2	28.0	17.5
Knowledge	0.0	34.8	0.0
Sports knowledge	2.2	13.6	0.0
Health-related knowledge	1.6	18.2	3.8
Self-awareness	20.4	1.5	7.5
Personal competence	15.1	7.6	2.5
Attitudes/enjoyment	18.3	5.3	12.5
Total	46.7%	33.2%	20.1%

#### Cluster 1—“Enjoyment-Oriented” (*n* = 142)

Students in this group emphasised enjoyment of physical and sporting activities (62.0%) and the importance of maintaining an active lifestyle (58.5%). Lower proportions associated physical literacy with self-awareness (27.5%) or personal competence (18.3%). This cluster portrays a conception centred on affective engagement and participation rather than performance or skill-based dimensions.

#### Cluster 2—“Performance-Focused” (*n* = 168)

Participants in this profile related physical literacy primarily to motor competence (61.9%), physical fitness (56.5%), sports competence (48.8%), and knowledge-related categories (e.g., sports knowledge, health knowledge; 35–42%). Although this group covered a wider range of categories, its responses were less concentrated, reflecting a performance- and knowledge-based conception but with greater dispersion in emphasis.

#### Cluster 3—“Competence-Centred” (*n* = 87)

This cluster displayed a highly consistent pattern, with responses concentrated in motor competence (74.7%) and active lifestyle (68.9%), followed by attitudes/enjoyment (41.4%). Students in this group expressed more coherent and narrowly defined ideas about physical literacy, integrating competence with behavioral engagement.

### Multinomial logistic regression

#### Cluster 3—“Competence-Centred” (*n* = 87)

A multinomial logistic regression was conducted to determine which contextual variables predicted membership in each of the three clusters, using the “Enjoyment-Oriented” cluster as the reference category. Table 4 shows the likelihood ratio tests of the regression performed with the contextual variables. In this case, the variables that determine membership of each cluster are associated with lifestyle. Specifically, the level of physical activity (measured in hours of sports or physical activity), performing a sports or physical activity before and during the training period and whether the sport in question was of a federated or recreational nature. In addition to the lifestyle variables, the academic year they were in at the time of completing the questionnaire was also a determining factor of cluster membership, as well as other biographical factors such as the mothers’ level of education. Table 4 presents the goodness-of-fit of the regression model indicating an adequate overall fit: −2 Log Likelihood = 612.45, McFadden’s *R*^2^ = 0.18, Cox & Snell *R*^2^ = 0.32, Nagelkerke *R*^2^ = 0.37, and classification accuracy = 68.4%. The goodness-of-fit tests (Pearson *χ*^2^ = 857.32, df = 820, *p* = 0.41; Deviance *χ*^2^ = 795.11, df = 820, *p* = 0.62) were non-significant, confirming that the model adequately fits the data.

**Table 4:** Likelihood ratio test.

Effects	Likelihood ratio test	
	*χ* ^2^	*p*
*Intersection*	0.000	.
Hours of sports activity per week	10.355	0.006*
IMC	0.643	0.725
University	2.833	0.243
Academic year	11.467	0.022*
Gender	2.317	0.314
Father’s academic level	5.845	0.441
Father’s occupation	10.449	0.107
Mother’s academic level	18.164	0.006*
Mother’s occupation	7.885	0.247
Previous experiences in teaching SPA	3.402	0.493
He/she is currently working as a trainer	2.059	0.725
He/she regularly did physical activity or sport before his/her studies	18.906	0.000*
Federated/recreational sport	18.021	0.005*
He/she currently does SPA	12.580	0.014*
Classification of PE	1.023	0.600
Academic level in higher education	9.026	0.172
Academic level in primary school	12.302	0.056
Academic level in secondary education	11.120	0.085
Academic level of PE in primary education	5.243	0.513
Academic level of PE in secondary education	4.363	0.359
*Goodness-of-fit coefficient*	736,273	0.118

*Statistically significant items; *χ*^2^, chi-square; *p*, *p* value; BMI, Body Max Index; SPA, sport or physical activity; PE, Physical Education.

These findings indicate that students’ conceptions of physical literacy are influenced by both lifestyle-related variables (e.g., activity level, sport type) and biographical factors (e.g., education level, training stage), confirming the predictive relevance of experiential and socialisation contexts.

### Longitudinal evolution

Table 5 shows how the ideas of future PE teachers regarding what they consider to be a physically literate student has evolved over the 5 years of their initial training process. The table shows the students’ affirmative responses to each of the categories, as in previous tables and due to the content of the research question, the same student may have responded to several categories. At the beginning of their training, the sample considered that a physically literate student was one who maintained an active lifestyle, had general knowledge of the subject, enjoyed doing physical and sporting activities and was personally competent. However, at the end of the first stage of their education process, students came to regard a physically literate learner as one who was mainly motor competent, enjoyed an active lifestyle and was self-aware. In addition to the change in the learner’s category preferences, a greater unanimity can also be observed in the responses, showing higher percentages and fewer being chosen. Ultimately, and after completing the training process (end of the master’s degree), the students considered a physically literate student to motor-competent, self-aware, and having an active lifestyle. Once again, the percentages increase and the categories of choice decrease.

**Table 5:** Comparison of student responses according to course.

	First year		Last year		Master		*χ* ^2^	*p*
	*n*	%	*n*	%	*n*	%		
Overall motor competence	7	14.0	14	40.0	5	50.0	9.881	0.007*
Sport competence	1	2.0	3	8.6	1	10.0	2.286	0.319
Physical fitness	2	4.0	0	0.0	0	0.0	1.839	0.399
Physical competence	6	12.0	0	0.0	1	10.0	4.457	0.108
Sports-related physical fitness	1	2.0	0	0.0	0	0.0	0.910	0.635
Health-related physical fitness	0	0.0	0	0.0	0	0.0	–	–
Active lifestyle	12	24.0	12	34.3	2	20.0	1.401	0.496
Knowledge	10	20.0	2	5.7	0	0.0	5.423	0.066
Sports knowledge	4	8.0	1	2.9	0	0.0	1.713	0.425
Health-related knowledge	3	6.0	3	8.6	0	0.0	0.984	0.612
Self-awareness	6	12.0	4	11.4	3	30.0	2.525	0.283
Personal competence	10	20.0	0	0.0	0	0.0	10.059	0.007*
Attitudes/enjoyment	10	20.0	1	2.9	1	10.	5.553	0.062

*Statistically significant items; *χ*^2^, chi-square; *p*, *p* value; *n*, students who answered the indicated category.

The category of motor competence, in addition to the observation made regarding the percentages and frequencies, displays statistically significant differences, showing a progressive increase in the importance that the students give to this category as their years of training expand. The opposite case is observed with the category of personal competence, which displays statistically significant differences since, at the beginning of their training, the students considered personal competence as a characteristic of physically literate students, and at the completion of their studies they discard this category.

These results indicate a linear and progressive shift from affective and personal dimensions (enjoyment, personal competence) toward more performance- and competence-oriented conceptions (motor competence, active lifestyle) as students advance through their training. The pattern was consistent across the three clusters, though the shift was strongest within the Competence-Centred group.

Taken together, the findings reveal a coherent developmental pattern. The cluster analysis identified three distinct conceptual profiles among future PE teachers. The regression model demonstrated that these profiles are shaped by students’ physical activity habits, prior experiences, and biographical context. The longitudinal analysis confirmed a progressive reorientation of conceptions toward performance and competence dimensions as training advances. Collectively, these results provide a comprehensive understanding of how conceptions of physical literacy are structured, influenced, and evolve throughout the initial teacher education process.

## Discussion

The objectives of this study were to establish profiles of trainee teachers’ ideas about physical literacy and to identify the role of initial teacher training in the evolution of their ideas about physical literacy. Teachers are the pillar on which educational transformations are based (Rupérez, 2021). For this reason, an approach to teachers’ conceptions of Physical Literacy can contribute to increasing the amount of research in this field (Durden-Myers and Keegan, 2019) and to stablishing the foundations of Physical Literacy in initial training.

Overall, the analysis of the results revealed three distinct profiles of ideas about what trainees believe a physically literate student should be. Although none of the profiles described are fully in line with the ‘Whitehedian’ concept (Edwards et al., 2018), the first of these is arguably the closest.

The Enjoyment-Oriented cluster, which emphasised enjoyment of physical activity (62%) and the importance of an active lifestyle (59%), aligns most closely with the affective and behavioral dimensions of Whitehead’s (2019) holistic conceptualisation of physical literacy. These participants appear to associate literacy with personal engagement, intrinsic motivation, and the pleasure derived from movement—elements considered central to developing lifelong participation in physical activity (Durden-Myers et al., 2018b). In contrast, the Performance-Focused cluster (42% of the sample) prioritised motor competence (62%), physical fitness (57%), and sports-related knowledge (35–42%), reflecting a more instrumental and performance-based conception. This orientation is consistent with traditional paradigms of physical education described by Fernández-Río et al. (2016), which privilege skill acquisition and measurable performance outcomes. Finally, the Competence-Centred cluster (22% of the sample) integrated motor competence (75%) with active lifestyle (69%) and enjoyment (41%), presenting a more coherent and mature understanding that links ability with engagement. This profile resonates with contemporary frameworks that view physical competence as a central but not exclusive component of physical literacy (Cairney et al., 2019; Lodewyk, 2019).

Thus, in the opinion of the students included in cluster 1, the importance of doing and enjoying physical and sports activity on a regular basis should be highlighted (Whitehead, 2019). Their opinions seem to show a line of thinking geared towards personal development and learning. These types of ideas are like definitions of teacher identity that relate to professional development and reflective practice (Imbernón, 2011; Shearer et al., 2018; Chan-Arceo and Canto-Herrera, 2022).

For the opinions of the second cluster, attributes related to physical and sporting skills are important and those related to personal development, or the enjoyment of physical and sporting activities are less important. This type of ideas, according to Fernández-Río et al. (2016), represents the more traditional versions of Physical Education, which focus on sports culture and the development of physical skills. However, there are some definitions and approaches to physical literacy that value and understand the importance of motor competence and physical activity as health promoters (Dudley, 2015; Cairney et al., 2019).

Situated halfway between the two previous proposals is the third profile, corresponding to the group of students that considers the main attribute of physical literacy to be physical competence and, secondarily, an active lifestyle and enjoyment of physical and sports activities. Considering motor competence as the main attribute of physical literacy is a recurring theme in literature on the concept, as several authors consider motor competence as the most salient aspect of physical literacy (Dudley, 2015; Lodewyk and Mandigo, 2017; Gu et al., 2019; Lodewyk, 2019). This may be because physical competence is the easiest element to quantify, score and assess. In fact, assessment is one of the handicaps that physical literacy must still address, according to Corbin (2016) and Lundvall (2015). Furthermore, Landi et al. (2021) consider that a renewal of the curriculum is necessary to adapt to such learner individualization. In this way, the three profiles share the importance of doing physical and sports activities regularly, coinciding with the study by Corbin (2016) in pointing out that this is one of the common focuses among the different institutions and authors that address the issue of physical literacy.

Similarly, there has also been a tendency to consider knowledge as a secondary attribute. However, some authors stress that cognitive aspects should be as important as physical ones (Sprake and Walker, 2015; Cale and Harris, 2018; Whitehead, 2019). These results might reflect the complexity of capturing the holistic and multidimensional essence of physical literacy. This may be due to the dualistic view, which separates the body from the mind and automatically directs thinking towards the physical dimension when it comes to concepts related to body and movement (Pot et al., 2018).

The multinomial regression analysis further clarified the contextual and experiential factors associated with these conceptual orientations. Among the different elements that appear to shape future teachers’ ideas, engagement in physical or sporting activities emerged as particularly significant. Specifically, higher weekly physical activity, previous competitive sport experience, and more advanced stages of training were associated with membership in the Competence-Centred and Performance-Focused clusters. In other words, depending on both the amount (number of hours) and the nature of the activity (recreational or competitive), the characteristics that students attribute to a physically literate learner vary. This finding aligns with previous research highlighting the influence of personal experiences as learners on the formation of teacher identity (Öhman et al., 2001; Alves et al., 2019; Jarrett and Light, 2019; McEvoy et al., 2019). While evidence concerning the active influence of broader lifestyle habits remains more limited (Bolívar, 2007). Similarly, biographical factors such as gender, family teaching background, or prior teaching experience have been shown to play a role in shaping teacher identity (Lamote and Engels, 2010). However, given the correlational nature of the present design, these associations should not be interpreted as causal; it is plausible that both training exposure and prior personal experiences reinforce existing conceptions rather than producing unidirectional change.

The longitudinal results indicate a clear evolution of conceptions over time, with significant increases in the proportion of students who identified motor competence as central to physical literacy. The training models in many institutions have been referred to by Tinning (1996) as performance-oriented discourses. This transformation of thinking may be determined by the very orientation of initial training, since the training model observed in this study responds to a Comprehensive sports model, typical of the 70s and 80s, rather than current pedagogical models (Fernandez-Rio et al., 2018), while other authors (Imbernón and Guerrero, 2018) consider that this type of training model does not respond to the current needs of Physical Education students and advocate the necessity to find a new approach. In addition to the orientation towards physical skills and sports performance observed as students’ progress in their training, a tendency towards homogeneity of responses has also been observed. Prior to training, the sample showed more varied responses, and at the end of their studies these responses are grouped around motor competence and active lifestyle. This observation opens a debate about whether a passive transfer of knowledge is taking place, in which students do not develop a critical view and therefore do not become reflective practitioners (Sinclair and Thornton, 2018). Some authors consider that, in adult learning processes, adults should be active agents during the development of their learning by maintaining continuous training (Sato and Haegele, 2017). For their part, Carreiro Da Costa et al. (2016) state the need for teachers to develop a critical vision that allows them to promote the changes and evolutions necessary for the improvement of education. It is through reflective professionals, who seek new ideas with which to challenge the established models, that changes and improvements in education begin (Flemons et al., 2018). Martins et al. (2021) consider that, given the complexity of the concept of physical literacy, there is a need to create a physical literacy framework in non-English speaking countries to foster key competences that help people to maintain an active and healthy lifestyle.

Considering the limitations of the study, such as a deeper understanding of the ratings given, and the lack of a female sample that could provide information on the gender gap in physical literacy, it would be interesting to be able to use other research techniques, such as interviews, or more qualitative methodologies, such as case studies. In this way, it would be possible to gain a broader insight into the future teachers’ ideas, as well as into the evolution of their ideas. In the same way, it would be interesting to progress in the comparative analysis between the thinking of students in training and that of the teachers responsible for this training to provide a more thorough analysis of how training courses influence students’ conceptions.

## Conclusion

This study identified three coherent profiles of conceptions about physical literacy among pre-service physical education teachers and documented a progressive shift from enjoyment- and participation-oriented perspectives toward more competence- and performance-oriented views throughout training. Although this trend appears temporally associated with teacher education, the correlational and observational nature of the design precludes causal inference. The longitudinal approach allowed for the observation of developmental and contextual changes in students’ thinking without the confounding effects of cross-sectional differences, revealing a gradual convergence of ideas around motor competence and active lifestyle attributes by the end of training.

Future research should employ qualitative or mixed-method approaches—such as interviews, focus groups, or longitudinal case studies—to examine how specific curricular components and pedagogical experiences shape these evolving conceptions. Comparative studies across institutions or national contexts would also clarify how different training models and cultural settings influence interpretations of physical literacy.

In sum, this study provides empirical evidence on how conceptions of physical literacy are structured and evolve during initial teacher education, highlighting both the progress made and the ongoing challenges in promoting a balanced and holistic understanding of physical literacy within teacher preparation programmes.

## Data Availability

The raw data supporting the conclusions of this article will be made available by the authors, without undue reservation.
