# Correction: Physical literacy conceptions in teacher training: a longitudinal study

**DOI:** 10.3389/fpsyg.2026.1813795

**Published:** 2026-03-05

**Authors:** Ariadna Hernaiz-Sánchez, Miguel A. González-Valeiro, Óscar Romero-Chouza, María A. Fernández-Villarino

**Affiliations:** 1 Departamento de Educación e Innovación Educativa, Facultad de Ciencias Jurídicas, Educación y Humanidades, Universidad Europea de Madrid, Campus de Villaviciosa, Madrid, Spain; 2 Department of Physical Education and Sport, University of A Coruña, A Coruña, Spain; 3 CIDEGA, Faculty of Education Sciences and Sport, University of Vigo, Pontevedra, Spain

**Keywords:** physical literacy, physical education, teacher training, beliefs and attitudes, higher education

An incorrect Funding statement was provided. The correct funder is Ministerio de Ciencia, Innovación y Universidades y Agencia Estatal de Investigación (MICIU/AEI). The correct funding statement reads:

“This publication was part of the project PID2021-128640OB-I00, funded by MICIU/AEI/10.13039/501100011033/ and by FEDER/EU.”

The original version of this article has been updated.

